# Novel Insights into the Roles and Mechanisms of GLP-1 Receptor Agonists against Aging-Related Diseases

**DOI:** 10.14336/AD.2021.0928

**Published:** 2022-04-01

**Authors:** Wei Peng, Rui Zhou, Ze-Fang Sun, Jia-Wei Long, Yong-Qiang Gong

**Affiliations:** ^1^Department of Gastrointestinal Surgery, Hunan Provincial People’s Hospital, The First Affiliated Hospital of Hunan Normal University, Changsha, Hunan, China; ^2^Hunan Normal University School of Medicine, Changsha, Hunan, China.

**Keywords:** glucagon-like peptide-1, GLP-1 receptor agonists, aging, aging-related diseases

## Abstract

Aging and aging-related diseases have emerged as increasingly severe health and social problems. Therefore, it is imperative to discover novel and effective therapeutics to delay the aging process and to manage aging-related diseases. Glucagon-like peptide-1 receptor agonists (GLP-1 RAs), one of the classes of antihyperglycemic drugs, have been recommended to manage type 2 diabetes mellitus (T2DM). Moreover, GLP-1 RAs have been shown to protect against oxidative stress, cellular senescence and chronic inflammation, which are widely accepted as the major risk factors of aging. However, their significance in aging or aging-related diseases has not been elucidated. Herein, we explain the underlying mechanisms and protective roles of GLP-1 RAs in aging from a molecular, cellular and phenotypic perspective. We provide novel insights into the broad prospect of GLP-1 RAs in preventing and treating aging-related diseases. Additionally, we highlight the gaps for further studies in clinical applications of GLP-1 RAs in aging-related diseases. This review forms a basis for further studies on the relationship between aging-related diseases and GLP-1 RAs.

## 1.Introduction

Aging due to molecular and cellular damage decreases the physical and mental capacity in a time-dependent manner. It is a significant risk factor for the higher incidences of mortality and morbidity associated with various chronic diseases [1]. The aging population is increasing worldwide and should be addressed. The number of people over 60 years is 1 billion based on the World Health Organization (WHO). Moreover, it is estimated that individuals over 60 years may possess 22% of the world’s population (about 2.1 billion) by 2050 [2]. The prevalence of aging-related diseases is becoming a growing concern due to the prolonged life expectancy and rapidly aging population. Aging-related diseases, such as type 2 diabetes mellitus (T2DM), renal function decline, cancers, etc., significantly affect the health and life quality of the elderly [1]. Aging-related diseases pose a great challenge to medical care due to the high incidence of senile diseases, poor treatment effects, and high coexistence rates of multiple diseases, thus bringing a substantial economic burden worldwide [3]. Therefore, new treatment strategies and drugs are needed for the management of aging-related diseases.

Glucagon-like peptide-1 receptor agonists (GLP-1 RAs), including native GLP-1 derivatives (albiglutide, dulaglutide, liraglutide, and semaglutide) and exendin-4 derivatives (exenatide and lixisenatide), trigger the release of insulin from the pancreas, inhibiting the secretion of glucagon from pancreatic α cells. Besides, GLP-1 RAs are used for the clinical treatment of T2DM [4]. Table 1 summarizes the pharmacokinetic and toxicological features of GLP-1 RAs. Besides their strong hypoglycemic effects, GLP-1 RAs can significantly reduce the risk of hypoglycemia and lower lipid levels, maintain blood pressure, and enhance cardiovascular protection and renal protection [5-9]. Therefore, GLP-1 RAs can be used to reduce polypharmacy in older adults, especially those with multimorbidity [5, 10]. A recent study showed that GLP-1 RAs might protect against chronic inflammation, oxidative stress, cellular senescence, etc., all of which are the major risk factors of aging [11-14]. Furthermore, various clinical trials have demonstrated that GLP-1 RAs play important roles in delaying and treating aging-related diseases.

This article reviews the function of GLP-1 RAs in delaying aging and ameliorating aging-related diseases, thus providing a theoretical basis and scientific guidance for clinical application of GLP-1 RAs.

**Table 1:** Pharmacokinetic and toxicological features of GLP-1 Ras.

Agents	Liraglutide	Dulaglutide	Albiglutide	Semaglutide	Exenatide	Lixisenatide
				Subcutaneous | Oral	Normal | Extended release	
T_1/2_	13h	5d	5d	1w	2.4h |/	3h
T_max_	8-12h	24-72h	3-5d	1-3d | 1h	2.1h | 2w, 6-7w	1-3.5h
Bioavailability	55%	65%, 47%	/	89%, 0.4%-1%	/	/
Protein binding	>98%	/	/	>99%	/	/
Volume of distribution	13L, 20-25L	19.2L, 17.4 L	11L	12.5L, 8L	28.3L	100L
Metabolism	A similar manner to large proteins without a specific organ as a major route of elimination	May be degraded into its component amino acids by general protein catabolism pathways	May be catabolized primarily in the vascular endothelium	Proteolytic cleavage of the peptide backbone and sequential beta-oxidation of the fatty acid sidechain	May through glomerular filtration and proteolytic degradation.	May through glomerular filtration and proteolytic degradation
Excretion	urine 6%, feces 5%	/	/	urine 3%, feces/	/	/
Effects of age on pharmacokinetics	None	None	None	None	None	None
Most common side effects	headache, nausea, diarrhea	nausea, abdominal pain, diarrhea, vomiting, decreased appetite	upper respiratory tract infection, back pain, diarrhea, joint pain, nausea, inflammation of the sinuses, reactions at injection site, flu symptoms, cough	nausea, vomiting, diarrhea, stomach pain, constipation	nausea, feeling jittery, indigestion, vomiting, dizziness, constipation, diarrhea, headache, weakness a bump at the injection site, nausea	nausea, vomiting, headache, diarrhea, feeling dizzy

## 2.Aging-related changes from different perspectives

**Figure 1.**
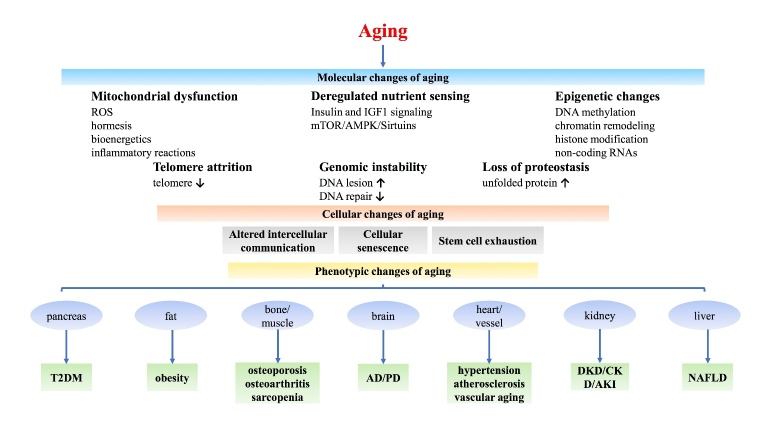
The mechanisms of aging.

Aging is highly complex progress associated with many biological and pathological changes. This part elucidates the underlying alterations and mechanisms in aging from different perspectives (Fig. 1).

### 2.1 Aging-related molecular and cellular changes

**Figure 2.**
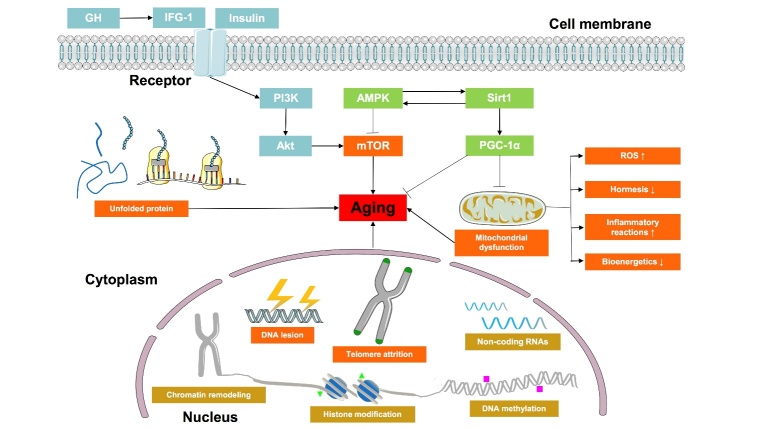
Molecular and cellular changes in aging.

Aging is associated with complex molecular and cellular changes (Fig. 2). Several biological hallmarks representing common aging characteristics, including epigenetic alteration, genomic instability, etc., have been systematically and comprehensively elucidated [1]. Genomic instability is caused by an imbalance between DNA damage and repair. Aging-related genomic damage is caused by both extrinsic and intrinsic factors [15]. Telomeres, repetitive DNA sequences, shorten every time cells divide. DNA damage signaling pathways and cellular senescence are triggered when telomeres are very short [16]. Epigenetic alterations, such as histone acetylation, chromatin remodeling, and DNA methylation, influence gene expression and genomic integrity and promote aging [17]. Non-coding RNAs mediate epigenetic modifications that may regulate aging and aging-related diseases [18]. The balance among protein synthesis, degradation and folding is key to maintaining protein homeostasis and thus ensuring longevity. Therefore, the loss of protein homeostasis leads to aging and diseases [19-21]. Moreover, insulin-like growth factor 1 (IGF-1), mammalian target of rapamycin (mTOR), adenosine 5’-monophosphate activated protein kinase (AMPK), and sirtuins-mediated metabolic signaling pathways are associated with the balance between nutrient anabolism and catabolism in cells, which maintain cellular homeostasis and play essential roles in aging [1, 22, 23]. Mitochondria are generally considered to be sources of cytotoxic reactive oxygen species (ROS). Many studies have suggested mitochondrial dysfunction is related to aging. Nevertheless, mitochondrial ROS are not always harmful and can even extend lifespan in mammals [24]. Therefore, the roles and mechanisms of mitochondrial dysfunction in aging should be further explored. Cellular senescence is characterized by secretory phenotype (SASP), macromolecular damage, altered metabolism, and irreversible cell-cycle withdrawal. It is involved in various biological processes and aging-related diseases [25]. Cellular senescence can be regarded as the positive compensatory response to injury. However, it may become detrimental and accelerate the aging process when the regenerative capacity of tissues is depleted [1]. Stem cell exhaustion causes a decline in tissue regeneration and significantly impacts the aging process. Moreover, the aging-related physiological decline can be protected by reducing stem cell senescence in the hypothalamus [26]. Furthermore, aging is often associated with changes in cell-to-cell communication, which are features of aging-related diseases, including Alzheimer’s disease (AD) and Parkinson’s disease (PD) [27]. Altogether, these biological hallmarks provide a better insight into the molecular and cellular changes in aging, building a framework for future studies.

### 2.2 Aging-related phenotypic changes

Molecular, phenotypic, and functional hierarchical domains establish the interlaced time and hierarchical relationship of aging. Aging is not a single cell death but a collection of phenotypes that respond to specific stimuli and follow particular dynamics. These specific aging processes are reflected in the physiological and pathological consequences of aging phenotypes. The elderly is predisposed to sarcopenia, osteoporosis, osteoarthritis, and fractures due to the progressive decline of muscle mass and strength, bone mineral density (BMD), and joint mobility. Endocrine imbalance and autonomic disorders cause dizziness, nausea, anxiety, and insomnia. Older people may also experience memory loss, mental retardation, cognitive impairment, and motor coordination deficits due to a gradual decline in the number and activity of neurons. Aging also increases arterial stiffness and decreases arterial elasticity and baroreceptor reflex sensitivity in the cardiovascular system, thus increasing blood pressure. Moreover, aging reduces the elasticity of the chest wall and lung tissue and pulmonary ventilation function in the respiratory system. Atrophy degeneration of the respiratory mucosa inhibits the removal of foreign bodies and bacterial defense, making individuals prone to respiratory infections. Aging is associated with poor digestion and absorption of nutrients, mainly due to tooth loss or loosening and atrophy of gastrointestinal mucosal. Many older adults suffer from constipation due to poor intestinal peristalsis. Renal aging is often accompanied by structural changes in the glomerulus, tubules, interstitium, and vasculature, and functional changes, including a decline in the estimated glomerular filtration rate (eGFR). These changes cause urinary disorders such as urinary frequency, urinary incontinence, or nocturia.

Herein, aging-related phenotypic changes, a multifaceted decline in histological structure and organismal function, and the corresponding susceptibility to aging-related diseases have been described.

## 3. The roles of GLP-1 RAs in aging

GLP-1 RAs play crucial roles in aging. Activation of the GLP-1 receptor will enhance the DNA repair through the stimulation of apurinic/apyrimidinic endonuclease 1 (APE1) expression [28]. GLP-1 alleviates H_2_O_2_-induced senescence, modulates the antioxidant defense system, and attenuates cellular senescence and DNA damage caused by a series of oxidative stress [11, 29]. GLP-1 also has a neuroprotective effect, promoting DNA repair in neurodegenerative diseases [29, 30]. Additionally, liraglutide can ameliorate mitochondrial dysfunction via the cyclic AMP (cAMP)/PKA pathway [31]. Exenatide can correct mitochondrial energy crisis, improve mitochondrial morphology, and normalize mitochondrial dynamics [32]. Liraglutide also safeguards cardiomyocytes against mitochondrial dysfunction mainly caused by interleukin-1β. In H9c2 cardiomyoblasts, activating the GLP-1 receptor can significantly suppress methylglyoxal-induced mitochondrial dysfunctions [33, 34]. Moreover, activation of the GLP-1 receptor protects against apoptosis and inhibits ROS production and inflammatory reaction through acting Sirt1 [30, 35]. GLP-1 protects cell apoptosis via the PI3K/Akt/mTOR signaling pathway [36]. Liraglutide protects β cells apoptosis through the AMPK/mTOR signaling pathway [37]. Liraglutide also ameliorates inflammation through the mTORC1 signaling pathway [38]. Furthermore, hepatocyte steatosis can also be inhibited by GLP-1 through inducing the response of unfolded protein [39]. DPP4-GLP-1 axis modulates cellular senescence through AMPK/SIRT1/FOXO3a pathway [14]. Collectively, these findings demonstrate a range of anti-aging effects of GLP-1 RAs.

## 4. The roles of GLP-1 RAs in aging-related diseases

**Figure 3.**
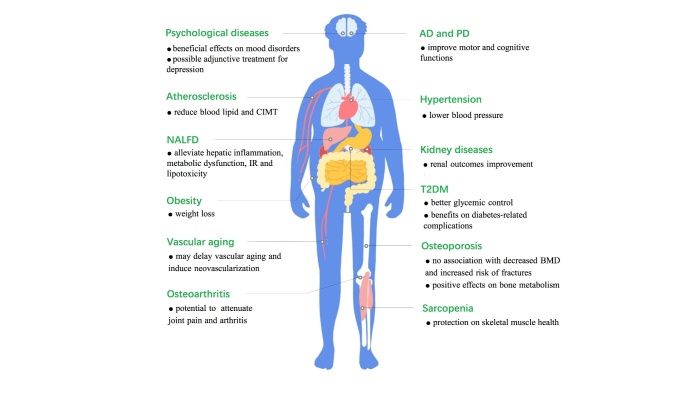
The roles of GLP-1 RAs in aging-related diseases.

Mounting evidence has shown that GLP-1 RAs are involved in various aging-related diseases. These include metabolic, neurodegenerative, cardiovascular, kidney, degenerative musculoskeletal diseases (Fig. 3). This section discusses the protective roles of GLP-1 RAs in aging-related diseases in cultured cells and animal models (Table 2 and 3). It focuses on the roles and clinical evidence of GLP-1 RAs in aging-related diseases in middle-aged and aged patients (Table 4).

**Table 2:** The roles of GLP-1 RAs in aging-related diseases in cultured cells.

Agents	Diseases	Cell types	Dosing	Duration	Effects	Ref.
GLP-1	Osteoporosis	Saos-2, TE-85, MG-63 cell lines	/	/	GLP-1 increases the viability levels of MG-63 and TE-85 osteoblastic cell lines.	[72]
GLP-1	Osteoporosis	Saos-2, TE-85 cell lines	/	/	GLP-1 affects bone metabolism possibly through the ATP-induced c-Fos activation.	[73]
GLP-1	Osteoporosis	ADSCs	10, 100 nM	/	GLP-1 stimulates osteoblast differentiation in ADSCs via ERK signaling pathway, whereas it inhibits adipocyte differentiation.	[75]
exendin-4	Alzheimer’s disease	SH-SY5Y cells, Primary neurons	0, 50, 100, 200, 500 nM	2h	Exendin-4 ameliorates the toxicity of Aβ and oxidative challenge in primary neuronal cultures and human SH-SY5Y cells in a concentration-dependent manner.	[89]
exenatide	Alzheimer’s disease	Brain ECs	5nmol/kg/day	4-5w	Exenatide strongly reverses aged mouse brain EC transcriptomic changes and BBB leakage.	[90]
GLP-1 exendin-4	Parkinson’s disease	SH-SY5Y cells	/	/	GLP-1 and exendin-4 stimulates cell proliferation and increased cell viability mainly via the PKA and PI3K signaling pathways.	[99]
GLP-1 exendin-4	Vascular aging	Human umbilical vein ECs	10 nmol/L	30min	GLP-1 prevents ROS-induced human umbilical vein endothelial cell senescence through the activation of PKA.	[11]
exendin-4	Vascular aging	VSMCs	/	/	Inhibiting Rac1 activation via a cAMP/PKA-dependent pathway and activating Nrf2 contribute to the protective effects of exendin-4 against ANG II-induced senescence in VSMCs.	[104, 105]
liraglutide	Atherosclerosis	Human THP-1 macrophages, bone marrow-derived macrophages	250 nmol/l	6h	Liraglutide decreases inflammatory response in MΦ0 THP-1 macrophages and bone marrow-derived macrophages	[117]
liraglutide	Atherosclerosis	VMSCs	100 nM / 1 μM	120h	Liraglutide may inhibit Ang II-induced VSMC proliferation by activating AMPK signaling and inducing cell cycle arrest, thus delaying the progression of atherosclerosis	[120]
exendin-4	Hypertension	LLC-PK1 cell line	1nM	30min	Exendin-4 regulates Na+/H+ exchanger NHE3 in renal proximal tubule cells.	[134]
exendin-4	Kidney diseases	HK-2 cells	0, 0.1, 1, 10, 100?nM	48h	Exendin-4 ameliorates high glucose-induced fibrosis by inhibiting the secretion of miR-192 from injured renal tubular epithelial cells.	[13]
exendin-4	Kidney diseases	MCs	0.1, 1, 10, 100nM	12,24,48 h	Exendin-4 alleviates high glucose-induced rat MC dysfunction through the AMPK pathway.	[147]
liraglutide	Osteoarthritis	Chondrocytes	100/500nM	24h	Liraglutide protects chondrocytes against endoplasmic reticulum stress and apoptosis induced by IL-1β or TGs.	[166]
liraglutide	Osteoarthritis	Human primary chondrocytes	50/100 nM	24h	Liraglutide suppresses TNF-α-induced degradation of extracellular matrix in human chondrocytes.	[167]
liraglutide	Sarcopenia	C2C12 cells	1μM	/	Liraglutide induces myogenesis in C2C12 myoblasts via a cAMP-dependent complex network of signaling events.	[168]
exendin-4	Sarcopenia	C2C12 cells	20 nM	60min/6h	Exendin-4 suppresses the expression of MSTN and muscle atrophic factors such as atrogin-1 and MuRF-1 in Dex-treated C2C12 myotubes.	[169]

ADSCs: adipose-derived stem cells; ANG: angiotensin; atrogin-1: F-box only protein 32; BBB: blood brain barrier; Dex: dexamethasone; ECs: endothelial cells; MCs: mesangial cells; MSTN: myostatin; MuRF-1: muscle RING-finger protein-1; TGs: triglycerides.

### 4.1 The roles of GLP-1 RAs in aging-related metabolic diseases

Aging is associated with many aging-related metabolic diseases, which cause disability and death. Compelling evidence has substantiated on the roles of GLP-1 RAs in aging-related metabolic diseases, such as T2DM, obesity, and osteoporosis.

#### 4.1.1 GLP-1 RAs and T2DM

Aging impairs β-cell function and reduces insulin sensitivity and secretion, thus predisposing T2DM development in the elderly [40, 41]. About 135.6 million people aged 65 to 99 years have diabetes globally, based on the latest data from the 9^th^ edition of the Diabetes Atlas by the International Diabetes Federation (IDF). Moreover, the number is expected to increase to 195.2 million by 2030 and 276.2 million by 2045 [42]. GLP-1 levels can also be reduced by aging in a fasting and glucose-stimulated state [43]. However, GLP-1 can reverse the age-associated impairment of insulin sensitivity and glucose tolerance [44, 45]. GLP-1 stimulates pancreatic cell proliferation and differentiation, thus improving pancreatic β-cell function in elderly rodents [46, 47]. Exendin-4 enhances the secretion of insulin from adult human pancreatic β cells [48]. Moreover, GLP-1 therapy improves pulsatile insulin secretion in elderly diabetic patients [49].

**Table 3:** The roles of GLP-1 RAs in aging-related diseases in animal models.

Agents	Models	Diseases	Dosing	Duration	Effects	Ref.
GLP-1	Wistar rats	T2DM	1.5 pmol/kg/min, s.c.	48 h	GLP-1 treatment can increase pancreatic insulin, GLUT2, and glucokinase mRNA in the old rats.	[44]
GLP-1	Wistar rats	T2DM	0.05, 0.1, 0.2, 0.4, 0.5 nmol/kg, IVGTT	-5, 0, 2, 4, 7, 10, 15, 20, 30 min	GLP-1 restores the acute insulin response to glucose and increases the clearance of glucose in the old animals.	[45]
GLP-1	Normal male mice	T2DM	25 nmol/kg/day, i.p.	12 d	GLP-1 counters aspects of the age-related impairment of pancreatic β-cell function and insulin sensitivity.	[46]
GLP-1	Wistar rats	T2DM	1.5 pmol/kg/min, s.c.	2 or 5 d	GLP-1 causes an up-regulation of PDX-1 expression in islets and total pancreas, induces pancreatic cell proliferation, and β-cell neogenesis.	[47]
exendin-4	NSG mice	T2DM	24 nmol/kg/day, s.c.	/	Exendin-4 stimulates insulin secretion by both juvenile and adult human β cells.	[48]
GLP-1	Adult male rats	Obesity	/	/	Intracerebroventricular (ICV) GLP-1 powerfully inhibits feeding in fasted rats.	[61]
exenatide	C57BL/6J mice	Obesity	24 nmol/kg, i.p.	8 w	Exenatide promotes brown remodelling of WAT in a SIRT1-dependent manner.	[66]
GLP-1 liraglutide exenatide	CD-1 mice, SD rats, cynomolgus monkeys	OP	/	/	GLP-1 RAs stimulate calcitonin release, up-regulation of calcitonin gene expression, and subsequently C-cell hyperplasia in rodents.	[78]
liraglutide	SAMP8 mice	AD	100 or 500 g/kg/day, s.c.	4 m	Liraglutide delays the progressive decline in memory function associated with hippocampal neuronal loss.	[86]
liraglutide	APP/PS1 mice	AD	25 nmol/kg	8 w	Liraglutide reduces inflammation response, β-amyloid plaque count, dense-core plaque numbers, soluble amyloid oligomers levels, prevents memory impairments, synapse loss and deterioration of synaptic plasticity, and increases young neurons numbers.	[87]
liraglutide	APP/PS1 mice	AD	25 nmol/kg, i.p.	2 m	Liraglutide can reverse some of the key pathological hallmarks of AD and prevents the progression of it.	[88]
exendin-4	3xTg-AD mice	AD	3.5 pM/kg/min, s.c.	16w	Exendin-4 reduces brain levels of tau, Aβ protein precursor and Aβ in STZ 3xTg-AD mice.	[89]
liraglutide lixisenatide	C57/BL6 mice	PD	2.5, 25, 250 nmol/kg, i.p./ lixisenatide (25 nmol/kg, i.p.)	5min,30min,3h/ 3week	Liraglutide and lixisenatide can cross the BBB and enhance neurogenesis.	[97]
NLY01	C57BL6, hA53T α-synuclein transgenic, TLR-2 KO mice	PD	3 mg/kg s.c., twice weekly	/	NLY01 exerts neuroprotective effects via the direct prevention of microglial mediated conversion of astrocytes to an A1 neurotoxic phenotype.	[98]
exenatide	ApoE-/-mice	Vascular aging	5 µg/kg s.c., twice daily	12w	Exenatide ameliorated vascular aging induced by high-fat diet.	[106]
exenatide	C57BL/6J mice	Vascular aging	5 μg/kg/day, s.c.	14d	Exenatide prevents vascular senescence.	[108]
liraglutide	ApoE-/-mice	Atherosclerosis	300 µg/kg, s.c. twice daily	4w	Liraglutide inhibits progression of atherosclerotic plaque formation and enhances plaque stability.	[113]
liraglutide	ApoE-/- mice	Atherosclerosis	0.4 mg/kg/day, s.c.	9w	Liraglutide ameliorates atherogenesis through reducing serum AGEs levels and RAGE.	[114]
exendin-4	C57BL/6J mice	Atherosclerosis	300 pmol/kg/day 24 nmol/kg/day, s.c.	28d	Exendin-4 reduces monocyte/macrophage accumulation in the arterial wall by inhibiting the inflammatory response in macrophages.	[116]
liraglutide	ApoE-/- mice	Atherosclerosis	300 μg/kg/day, s.c.	6 or 4 w	Liraglutide regulates immune cell phenotypes in early and preestablished atherosclerosis.	[117, 118]
lixisenatide liraglutide	Apoe -/-Irs2 +/- mice	Atherosclerosis	lixisenatide (10 μg/kg/day, s.c) liraglutide (400 μg/kg/day, s.c)	1 m	Lixisenatide decreases atheroma plaque size and instability by reprogramming macrophages towards an M2 phenotype.	[119]
liraglutide	ApoE-/-mice	Atherosclerosis	400 μg/day, s.c.	4 w	Liraglutide suppresses atherosclerotic lesions and increases AMPK phosphorylation in the aortic wall.	[120]
GLP-1	SD rats	Hypertension	30 pmol/kg/min, IVGTT	120 or 30min	GLP-1 acutely recruits microvasculature and increases basal glucose uptake in muscle via a NO-dependent mechanism	[129]
liraglutide	C57BL/6 mice	Hypertension	27or30 μg/kg, i.p. twice daily	/	Liraglutide promotes vasorelaxation by inducing the secretion of ANP.	[130]
GLP-1	Wistar rats	Hypertension	1 μg/kg·min, i.p.	60min	GLP-1 can exert direct effects on relaxing rat conduit arteries, independently of NO and the endothelium.	[133]
liraglutide	SHR, WKY rats	Hypertension	0.9?µg/3?µl/day, i.v.	15d	Liraglutide attenuates the progression of hypertension in SHR through activating brainstem DBH neurons and suppressing sympathetic nerve activity.	[136]
liraglutide	STZ diabetic rats	Kidney diseases	0.3 mg/kg/12 h, s.c.	4w	Liraglutide against oxidative stress and diabetic nephropathy via a PKA-mediated inhibition of renal NAD(P)H oxidase.	[12]
liraglutide	Wistar rats	OA	50?μg/kg/day, s.c.	28d	Liraglutide ameliorates inflammation through the activation of the PKA/CREB pathway in OA rats.	[165]
liraglutide	SD rats	Sarcopenia	200µg/kg, s.c. twice daily	/	Liraglutide ameliorates skeletal muscle atrophy in rodents.	[168]
exendin-4 dulaglutide	C57BL/6, DBA/2J-mdx mice	Sarcopenia	exendin-4 (100 ng/day, s.c.) dulaglutide (1 mg/kg/week, s.c)	exendin-4(8w) dulaglutide(3w)	GLP-1 RAs ameliorate muscle wasting by suppressing MSTN and muscle atrophic factors and enhancing myogenic factors.	[169]

AGEs: advanced glycation end products; DBH: dopamine beta-hydroxylase; MSTN: myostatin; OA: osteoarthritis; OP: osteoporosis; RAGE: receptor for advanced glycation end products; SD: Sprague-Dawley; SHR: spontaneously hypertensive rats; STZ: streptozotocin; WAT: white adipose tissue; WKY: Wister Kyoto rats

Lixisenatide is one of the most suitable GLP-1 RAs used for T2DM pathophysiology therapy in the elderly. A pooled analysis conducted on data from six GetGoal trials suggested that lixisenatide can effectively treat elderly patients with T2DM [50]. Moreover, lixisenatide, as an add-on to oral antidiabetics (OADs), can significantly improve glycemic control in T2DM patients aged ≥65 years [51]. Another post hoc analysis showed that lixisenatide combined with basal insulin could effectively treat T2DM in patients aged ≥70 years. Notably, the effectiveness and safety of lixisenatide are virtually not affected in moderate renal insufficiency patients [52]. Furthermore, oral semaglutide was recently approved for treating T2DM in elderly patients with or without comorbidities [53]. GLP-1 RAs have a lower risk of diabetic ketoacidosis, lower-limb amputations, and genital infections and similar major adverse cardiovascular events (MACE) risk among older adults compared with sodium-dependent glucose transporters 2 (SGLT2) inhibitors [54, 55]. Moreover, GLP-1 RAs combined with SGLT2 inhibitors positively impact systolic blood pressure (SBP), A1C level, and body weight in elderly T2DM patients [56].

**Table 4:** The roles of GLP-1 RAs in aging-related diseases in clinical trials.

Agents	Type of study	Sample information	Dosing	Findings	Ref.
T2DM					
lixisenatide	phase I, single-centre, open-label study	18 (≥65)/ 18 (18-45)	20 µg a single	Mean exposure and the terminal half-life was higher in elderly subjects; C(max), t(max) and adverse events were comparable in both groups	[50]
lixisenatide	pooled analysis	2565(<65)/ 623 (≥65)	20 µg, 12/24 m once daily	Lixisenatide significantly reduced HbA1c vs placebo in all age groups	[50]
lixisenatide plus OADs	meta-analysis	501 (≥65)	20 µg, 24 w once daily	Lixisenatide plus OADs improved glycemic control	[51]
lixisenatide plus basal insulin	post hoc analysis	108 (≥70)	20 µg, 24 w once daily	Lixisenatide significantly reduced HbA1c, 2-hour PPG, average seven-point SMPG, and body weight	[52]
oral semaglutide	review	/	/	Age did not affect glycemic efficacy of oral semaglutide	[53]
GLP-1 RAs	retrospective analysis	90094 (≥66)	/	GLP-1RAs had similar MACE risk, increased HHF risk, and decreased risk of DKA, LLA, and genital infections vs SGLT2i	[54]
GLP-1 RAs	systematic review and meta-analysis	93502 (≥65)	/	GLP-1 RAs reduced MACE	[55]
GLP-1 RAs plus SGLT2i	observational, prospective, multicenter study	113 (>65)	/	Combination therapy was tolerated well and reduced A1C level, body weight and SBP	[56]
Obesity					
semaglutide	STEP 2 trial	1210 (55.3±10.6)	2.4/1.0 mg, 14 w once weekly	Semaglutide (2.4mg) was associated with significant reduced bodyweight and higher incidence of adverse events	[6]
liraglutide	double-blind study	68 (58±8)	0.6-1.8 mg, 14 w once daily	Liraglutide plus calorie restriction significantly reduced weight and improved IR, SBP, glucose, and TG	[70]
liraglutide	perspective case series	9 (68.22±3.86)	3.0 mg, 24 w once daily	Liraglutide was associated with decrease in BMI, weight, fat mass and android fat	[71]
Osteoporosis					
exenatide	clinical trial	69 (59 ± 8)	/, 44 w,/	Exenatide had no adverse effects on BMD	[79]
GLP-1 RAs	cohort study	79964 (Mean: 55)	/	GLP-1 RAs were not significantly associated with increased risk for fracture	[81]
GLP-1 RAs	meta-analysis	4255 (56.4 ± 1.9)	/	GLP-1RA did not modify the risk of bone fracture	[82]
Alzheimer’s disease					
liraglutide	RCT	18(Mean:63.1)/ 20(Mean:66.6)/ 6(63±3)	0.6-1.8mg, 26 w once daily	Liraglutide significantly raised blood-brain glucose transfer and prevented the decrease of CMRglc	[91]
dulaglutide	exploratory analysis of the REWIND trial	9901 (≥50 y)	1.5 mg, median:5.4 y once weekly	Long-term use of dulaglutide may reduce cognitive impairment	[92]
liraglutide	RCT	38 (50-80)	0.6-1.8 mg, 26 w once daily	Liraglutide was associated with numerical increase in CMRglc, but no influence on in Aβ levels and cognitive scores	[93]
exenatide	RCT	21 (> 60)	5-10µg, 12 w twice daily	No differences in cortical thickness and volume, cognitive measures, or biomarkers between exenatide groups and placebo	[94]
liraglutide	RCT	43 (45-70)	0.6-1.8 mg, 12 w once daily	No detectable cognitive differences were found between liraglutide groups	[95]
Parkinson’s disease					
exenatide	RCT	20(61.4±6.0)/ 24 (59.4 ±8.4)	5-10μg, 12 m twice daily	Exenatide group showed a significant improvement in motor and cognitive functions, and these improvements persist for a long period after exenatide withdrawal	[100, 101]
exenatide	RCT	31(61.6±8.2)/ 29 (57.8 ±8.0)	2 mg, 48 w once weekly	Exenatide group showed improvements in practically defined off-medication motor scores	[102]
Atherosclerosis					
liraglutide	clinical trial	64 (63 ± 8)	0.6-1.2 mg, 8 m once daily	Liraglutide reduced TC, TG, LDL-C, and CIMT, whereas increased HDL-C	[7]
liraglutide	clinical trial	29(61 ± 10)/ 29 (61 ± 8)	0.6-1.2 mg, 8 m once daily	Liraglutide significantly reduced CIMT in patients with T2DM and NAFLD, but not in T2DM patients without NAFLD	[121]
liraglutide	clinical trial	121 (62 ± 9)	0.6-1.2 mg, 18 m once daily	Liraglutide significantly reduced waist circumference, BMI, fasting glycemia, HbA1c, TC, LDL-C, TG, and CIMT	[122]
Hypertension					
liraglutide	LEADER trial	9340 (≥50)	1.8mg, median:3.8 y once daily	Liraglutide decreased SBP by 1.2mmHg	[8]
semaglutide	SUSTAIN-6 trial	3297 (64.6±7.4)	0.5/1.0 mg, 104 w once weekly	The mean SBP in the semaglutide group was 1.3 mm Hg and 2.6 mm Hg lower in the group receiving 0.5 mg and 1.0 mg vs placebo, respectively	[125]
oral semaglutide	PIONEER 6 trial	3183 (≥50)	14mg, median:15.9 m once daily	The mean SBP in the oral semaglutide group was 2.6 mmHg lower	[126]
exenatide and liraglutide	meta-analysis	5860 (Mean: 55)	/	Exenatide and liraglutide reduced SBP and DBP by 1 to 5 mmHg vs some other hypoglycemic agents	[127]
GLP-1 RAs	systematic review and meta-analysis	26654 (Average:55.88)	/	GLP-1RAs were associated with modest reduction on BP, but no significant association with hypertension	[128]
Kidney diseases					
liraglutide	LEADER trial	9340 (≥50)	1.8mg, median:3.8 y once daily	Liraglutide was associated with significant reduction in new-onset severely increased albuminuria and lower rates of DKD events, but no association with increased risk of AKI	[8, 9]
exenatide plus insulin glargine	clinical trial	92 (56.0± 8.4)	5-10μg, 24 w twice daily	Exenatide plus insulin glargine significantly reduced albuminuria in patients with T2DM and DKD	[149]
dulaglutide	AWARD-7 trial	577 (64.7±8.8)/(64.7±8.6)/(64.3±8.4)	0.75/1.5mg, 52 w once weekly	Dulaglutide was associated with less eGFR decline vs insulin glargine	[151]
semaglutide	post-hoc analysis of the SUSTAIN 1-7 trials	8416 (Mean: 53.7-64.6)	0.5/1.0 mg, 30-104 w once weekly	Semaglutide was associated with initial reductions in eGFR and marked reductions in UACR, but no association with increased risk of kidney adverse events	[152]
dulaglutide	exploratory analysis of the REWIND trial	9901 (≥50)	1.5 mg, median: 5.4 y once weekly	Long-term use of dulaglutide reduced composite renal outcomes in people with T2DM	[153]
GLP-1RAs	systematic review and meta-analysis	56004 (Mean: 60-66)	/	GLP-1RAs conferred a reduction in a broad composite kidney outcome	[154]
GLP-1RAs	Scandinavian cohort study	38731 (59±10)	/	GLP-1 RAs reduced risk of serious renal events vs DPP4i	[155]
GLP-1RAs	systematic review and meta-analysis	77242 (Mean: 60-65)	/	GLP-1 RAs had a less marked effect on preventing hospitalization for progression of kidney disease vs SGLT2i	[157]
Sarcopenia					
liraglutide	perspective case series	9 (68.22±3.86)	3.0 mg, 12/24 m once daily	Liraglutide was associated with an improvement in SMI	[71]
NAFLD					
semaglutide	RCT	320 (Mean: 55)	0.1/0.2/0.4 mg, 72 w once daily	Semaglutide resulted in a significantly higher percentage of patients with NASH resolution vs placebo	[172]

AKI: acute kidney injury; DBP: diastolic blood pressure; DKD: diabetic kidney disease; BMD: Becker muscular dystrophy; BMI: body mass index; DPP4i: dipeptidyl peptidase 4 inhibitors; CIMT: constraint-induced movement therapy; CMRglc; cerebral metabolic rate for glucose; DKA: diabetic Ketoacidosis; eGFR: epidermal growth factor receptor; GLP-1 RAs: GLP-1 receptor agonists; HbA1c: glycated hemoglobin/hemoglobin A1c; HDL-C: high-density lipoprotein cholesterol; HHF: hospitalization for heart failure; IR: insulin resistance; LDL-C: low-density lipoprotein cholesterol; LLA: lower-limb amputations; MACE: major adverse cardiovascular events; NAFLD: non-alcoholic fatty liver disease; NASH: non-alcoholic steatohepatitis; OADs: oral antidiabetic drugs; PPG: prandial plasma glucose; RCT: randomized controlled trial; SBP: systolic blood pressure; SGLT2i: sodium-glucose cotransporter 2 inhibitors; SMPG: self-measured plasma glucose; T2DM: type 2 diabetes mellitus; TC: total cholesterol fasting blood glucose; TG: triglycerides; UACR: urinary albumin-creatinine ratio

#### 4.1.2 GLP-1 RAs and obesity

Aging is associated with gains in fat mass and the loss of muscle mass, which leads to obesity among older individuals [57]. Several factors regulate these aging-related changes in body compositions, including physical inactivity, resting metabolic rate reductions, hormone levels decline, and blunted lipolysis [57-60]. However, the precise mechanisms underlying the changes in body composition associated with aging are unknown. GLP-1 RAs regulate body fat accumulation and weight loss via three direct or indirect ways. First, GLP-1 RAs can regulate satiety and suppress appetite through the central nervous system. Intracerebroventricular GLP-1 treatment strongly inhibits food intake in fasted rats [61]. A recent crossover, randomized, placebo-controlled trial showed that liraglutide could affect the neural reaction to food cues in middle-aged T2DM patients [62]. Notably, GLP-1 receptors were firstly proved to exist in human brains [62]. Second, GLP-1 RAs influence gastrointestinal function by inhibiting gastric motility and delaying gastric emptying [63-65]. Third, GLP-1 RAs directly act on adipose tissue, promote brown remodeling of white adipose tissue and fat mobilization, accelerating fat burning and thus achieving sustained weight loss [66].

Liraglutide was approved by the U.S. Food and Drug Administration (FDA) (2014) for the treatment of obesity and chronic weight management in obese adolescents aged 12-17 [67, 68]. Studies have also assessed the safety and effectiveness of semaglutide in obese patients with or without T2DM for an enhanced therapeutic approach in the future [69]. However, there are few studies related to a weight loss of GLP-1 RAs focusing on older adults. A STEP 2 study showed that semaglutide significantly decreases the weight of T2DM patients with overweight or obesity (55.3±10.6 years) [6]. A clinical trial with 68 older adults with obesity and prediabetes found that liraglutide can significantly enhance weight loss. Moreover, liraglutide-mediated weight loss substantially improves insulin resistance, glucose tolerance, SBP, and triglyceride concentration. However, 79% of patients treated with liraglutide experienced gastrointestinal side effects [70]. Another prospective study with nine subjects demonstrated that a 24-week liraglutide treatment could effectively reduce fat mass in overweight and obese elderly with T2DM [71]. Overall, liraglutide can effectively promote and maintain weight loss in the elderly. However, further studies should assess the effect of semaglutide, a novel weight-loss therapeutic drug.

#### 4.1.3 GLP-1 RAs and osteoporosis

Aging reduces bone strength, thus increasing the risk of fracture (osteoporosis) in the elderly. The imbalance between bone resorption and bone formation during bone remodeling causes osteoporosis. GLP-1 treatment can improve the viability levels of MG-63 and TE-85 osteoblastic cell lines, suggesting that GLP-1 may bind to specific GLP-1 receptors on osteoblasts, thereby promoting bone formation [72]. GLP-1 influences bone metabolism, possibly through ATP-induced c-Fos activation [73]. Moreover, the expression of GLP-1 receptors is increased during the osteogenic differentiation of adipose-derived stem cells (ADSCs) [74]. GLP-1 stimulates the osteoblast differentiation and inhibits adipocyte differentiation in human ADSCs via ERK and Wnt/GSK-3β/β-catenin pathways [75, 76]. GLP-1 promotes the proliferation of human mesenchymal stem cells (hMSCs), reducing apoptosis and preventing their differentiation into adipocytes. Further evidence has shown that MEK and PKC pathways mediate the impacts of GLP-1 on these cells [77]. In addition, GLP-1 RAs may inhibit bone resorption by stimulating calcitonin release from thyroid C-cells [78].

A clinical study showed that exenatide treatment for 44 weeks does not affect BMD and serum markers of bone metabolism [79]. Another two-centered clinical trial reported no effect on BMD or bone turnover markers after 24 weeks of exenatide treatment in newly diagnosed T2DM patients [80]. Furthermore, GLP-1 RAs are not correlated to the increase in bone fracture risk [81]. Similarly, a meta-analysis showed that GLP-1 RAs are not related to fracture risk [82]. The level of serum GLP-1 decrease in older patients with fractures. However, vitamin D is positively correlated with GLP-1, suggesting GLP-1 has a bone-protective effect [83].

### 4.2 The roles of GLP-1 RAs in aging-related neurodegenerative diseases

Neurodegenerative diseases are caused by the loss of neurons or myelin sheaths, which deteriorate over time, resulting in dysfunction. GLP-1RAs play an essential role in aging-related neurodegenerative diseases.

#### 4.2.1 GLP-1 RAs and AD

AD is characterized by the deficits in cognition, memory, and learning. Emerging evidence shows that GLP-1 RAs are associated with AD. Impaired brain insulin signaling in T2DM is closely related to AD pathogenesis [84]. Exendin-4 can protect against apoptosis and regulate GLP-1/insulin/IGF-1 pathway in middle-aged T2DM rat brains [85]. Liraglutide can significantly increase hippocampal CA1 pyramidal neuron numbers and improve memory function in SAMP8 mice [86]. Liraglutide can also cross the blood-brain barrier (BBB) of 7-month-old APP/PS1 mice, reducing amyloid plaque, decreasing the inflammation response, and increasing young neurons [87]. Similar results have also been obtained in older APP/PS1 mice after three years [88]. The above two studies demonstrate that liraglutide has protective effects in the early and late AD stages. Beside reducing the levels of amyloid-beta (Aβ) protein precursor and Aβ in 3xTg-AD mice brains, exendin-4 can ameliorate Aβ toxicity and oxidative damage at the cellular level [89]. Moreover, exenatide can also significantly reverse transcriptomic alterations underlying brain endothelial cells (ECs) aging at a molecular level [90].

Liraglutide treatment can significantly increase blood-brain glucose metastasis and prevent a decrease in cerebral metabolic rate for glucose (CMRglc) in AD patients compared with placebo [91]. An exploratory analysis suggested that long-term treatment with dulaglutide has beneficial effects on cognitive impairment in patients aged ≥50 years with established or newly diagnosed T2DM [92]. Moreover, a 6-month liraglutide treatment increases CMRglc in AD patients but does not affect Aβ levels and cognitive scores [93]. An 18-month phase II clinical trial showed that exenatide treatment reduces Aβ42 levels in plasma neuronal extracellular vesicles (EVs). However, no significance in cortical thickness and volume, cognitive measures, or biomarkers were found between the exenatide group or placebo [94]. Meanwhile, it is unknown whether liraglutide has neuroprotective benefits in middle-aged persons with AD [95]. A multi-center study with 206 patients randomized to liraglutide is currently underway [96], and better results are highly expected in future research.

#### 4.2.2 GLP-1 RAs and PD

PD is shared among the elderly. Liraglutide and lixisenatide can cross the BBB, thus enhancing neurogenesis [97]. NLY01, a brain-penetrant GLP-1 RA, exerts neuroprotective effects by blocking the microglial-mediated transition of astrocytes into A1 neurotoxic phenotypes in PD mice [98]. Furthermore, GLP-1 has neurotrophic effects of protecting against human neuronal apoptosis at the cellular level [99]. Altogether, these results point to the function of GLP-1 RAs in treating PD.

Clinical evidence suggest that a 12 month-exenatide treatment can significantly improve motor and cognitive functions in PD patients [100]. Moreover, the motor and cognitive advantages could persist for an extended period after exenatide withdrawal [101]. Exenatide also improves practically defined off-medication motor scores in patients with moderate PD. However, it is unknown whether exenatide treatments can alter the course of the underlying PD [102]. A systematic review also suggested the function of exenatide in the improvement of motor impairment for PD patients requires deep research [103]. Hence, exenatide may represent a new therapeutic drug for treating PD, but additional studies are needed to evaluate its long-term effecacy in treating everyday symptoms.

### 4.3 The roles of GLP-1 RAs in aging-related cardiovascular diseases

Aging represents a crucial risk factor in the occurrence, development and outcome of cardiovascular diseases. GLP-1 RAs can benefit aging-related cardiovascular diseases, such as vascular aging, atherosclerosis, and hypertension.

#### 4.3.1 GLP-1 RAs and vascular aging

Experimental and clinical studies have shown that GLP-1 RAs decrease the risk of adverse cardiovascular events. However, the detailed mechanisms involved are unknown. Vascular aging contributes to the pathology of cardiovascular diseases. GLP-1/exendin-4 can attenuate ROS-induced ECs senescence via an AMP/PKA-dependent pathway [11]. Exendin-4 can also significantly alleviate angiotensin (ANG) II-induced superoxide generation and the subsequent vascular smooth muscle cells (VSMCs) senescence by targeting Rac1 and Nrf2 [104, 105]. Exenatide can ameliorate vascular aging stimulated by a high-fat diet in ApoE-/- mice by modulating inflammation and oxidative stress response [106]. Moreover, exenatide can protect endothelial dysfunction caused by ischemia-reperfusion injury in humans by opening K ATP channels [107]. Moreover, the dipeptidyl peptidase 4 (DPP-4)/GLP-1 axis can prevent vascular aging and maintain ischemia-induced neovascularization in mice [11, 108]. In ApoE-/- mice, increased DPP-4 levels promote diet-associated vascular aging in the presence of chronic stress [109]. Moreover, inhibiting DPP-4 would ameliorate chronic stress-related vascular aging, potentially through the improvements of oxidative stress and vascular inflammation [110]. DPP-4, mediated by the GLP-1/GLP-1R axis, can also regulate chronic stress-associated inflammatory cell production and bone marrow hematopoietic stem cell activation [111]. Overall, these findings imply a regulatory mechanism of GLP-1 RAs in vascular aging and indirectly suggest roles of GLP-1 RAs in managing cardiovascular diseases.

#### 4.3.2 GLP-1 RAs and atherosclerosis

Atherosclerosis is common in the elderly. Aging alters the vascular structure and function, such as intimal thickening, inflammation, and lipid deposition, thus accelerating the progression of atherosclerotic diseases [112]. However, liraglutide can inhibit the formation of atherosclerotic plaques and enhance the stability of early atherosclerotic plaques by binding to the GLP-1 receptor [113]. Liraglutide can also ameliorate atherogenesis by reducing serum-advanced glycation end products (AGEs) expression of receptor for advanced glycation end products (RAGE) [114]. DPP-4 inhibition decreases chronic stress-related carotid artery thrombosis, possibly by improving oxidative stress [115]. DPP4 inhibition can also attenuate oxidative stress, plaque inflammation, and proteolysis related with GLP-1-mediated adiponectin production [109]. Monocytes and macrophages, primary immune cells, are involved in the inflammatory processes in the atherosclerotic lesion. GLP-1 RAs induce anti-atherosclerotic effects by reducing monocyte/macrophage accumulation in the arterial vessel, regulating pro-inflammatory mediators, and modulating immune cell phenotypes [116-119]. Moreover, liraglutide enhances bone marrow-derived macrophages in mice and MΦ2 phenotypes in human THP-1 [117]. Liraglutide can also inhibit VSMCs proliferation through AMPK signaling activation and cell cycle regulation, thereby delaying atherosclerosis [120].

A prospective study showed that an 8-month liragluide treatment could reduce triglycerides (TG), low-density lipoprotein-cholesterol (LDL-C), and total cholesterol (TC), but increase high-density lipoprotein cholesterol (HDL-C) in T2DM patients [7]. The treatment can also significantly decrease carotid intima-media thickness (CIMT), a surrogate marker of subclinical atherosclerosis [7, 121]. Liraglutide can significantly reduce LDL-C, TG, and CIMT levels in T2DM and metabolic syndrome [122]. Altogether, these studies indicate that GLP-1 RAs are strongly associated with hyperlipidemia and atherosclerosis, especially in middle-aged and older patients.

#### 4.3.3 GLP-1 RAs and hypertension

Hypertension is ubiquitous in the elderly, and more than half of people aged 45-75 years in China and the United States suffer from hypertension [123]. Liraglutide combined with OADs, such as rosiglitazone, glimepiride, and metformin have antihypertensive effects [124]. Several recent studies have investigated whether GLP-1 RAs alone can exert blood-pressure-lowering effects. Liraglutide can decrease SBP by 1.2 mmHg [8]. In SUSTAIN-6 trial, respectively, the mean SBP in the semaglutide group receiving 0.5 mg and 1.0 mg was 1.3 mm Hg and 2.6 mm Hg lower than the placebo [125]. Similarly, compared with placebo group, the SBP was 2.6 mmHg lower in the oral semaglutide group in the PIONEER 6 trial [126]. Moreover, considerable evidence supports that GLP-1 RAs are also associated with a modest reduction in diastolic blood pressure (DBP) in middle-aged and older patients with T2DM [127, 128].

The principal mechanisms of antihypertensive effect of GLP-1 RAs may consist of the following four fronts. First, GLP-1 RAs promote vasodilation. Chai *et al*. found that GLP-1 enhances muscle microvascular blood volume (MBV) in overnight-fasted adult male rats by increasing the production of nitric oxide (NO) [129]. Kim *et al*. also indicated that liraglutide induces atrial natriuretic peptide (ANP) secretion, thereby promoting vasorelaxation [130]. Notably, GLP-1 can directly affect relaxing rat conduit arteries, independently of NO and the endothelium [131]. GLP-1 infusion can regulate vasorelaxation in the brachial artery of middle-aged patients with T2DM and coronary artery disease [132]. Second, GLP-1 RAs facilitate and promote natriuresis. GLP-1 receptor can be expressed on atrial cardiomyocytes. GLP-1 RAs can increase cAMP via activating GLP-1 receptor and mediate ANP release, leading to urine sodium excretion and blood pressure (BP) reduction [130]. GLP-1 RAs may also increase urinary sodium excretion by regulating Na^+^/H^+^ exchanger in renal tubules [133, 134]. Third, GLP-1 RAs indirectly control BP through weight loss. Weight loss is correlated with BP reduction in middle-aged and older T2DM patients [135]. Fourth, GLP-1 RAs are involved in the central control of BP. Liraglutide attenuates the progression of hypertension in spontaneously hypertensive rats by activating brainstem dopamine beta-hydroxylase (DBH) neurons and suppressing sympathetic nerve activity [136].

### 4.4 The roles of GLP-1 RAs in aging-related kidney diseases

Aging alters the kidney structure, such as decreased renal mass and cortical thickness, glomerulosclerosis, glomerular basement membrane (GBM) thickening, interstitial fibrosis, and tubular atrophy [137]. The eGFR declines by about 5%-10% per decade after 30 years [138], one of the most predominant functional changes of renal aging. It is usually difficult to distinguish common kidney diseases in the elderly, such as diabetic kidney disease (DKD), acute kidney injury (AKI), and chronic kidney disease (CKD), which are caused by normal aging or are secondary to the disease development. However, aging is undoubtedly a vital part of the pathogenesis of these kidney diseases in the elderly [137, 139, 140]. GLP-1 RAs have been used by almost all clinical trials for kidney outcomes in the diabetic population, the majority of whom are elderly. Herein, we differatiate between patients with DKD and those with diabetes and CKD.

Accelerated kidney aging promotes DKD progression [139]. Kidney aging in diabetic individuals involves cellular senescence, AGEs accumulation, renin-angiotensin-aldosterone system (RAAS) dysfunction, inflammation, and oxidative stress [139, 141-143]. Moreover, downregulation of anti-aging gene *klotho* and *Sirt1* and the impairment of lysosome-dependent autophagy promote DKD development [139, 144-146]. Several pre-clinical research pointed out that GLP-1 RAs can ameliorate renal injury induced by aging and diabetes by reducing renal inflammation, relieving renal oxidative stress, and inhibiting renal fibrosis [12, 13, 147]. DPP-4 inhibition exerts protective roles on podocyte injury through antioxidant and anti-apoptotic mechanisms [148]. A LEADER trial indicated that liraglutide significantly reduces new-onset severely increased albuminuria compared with placebo. Liraglutide is also associated with lower rates of DKD events than placebo [9]. A small clinical trial conducted in China assessed the renal outcomes of exenatide in T2DM and DKD patients (mean age, 56 years). The inclusion criteria were an eGFR ≥30 mL/min/1.73m^2^ and 24h urinary albumin excretion rate (UAER) >0.3g/24h. Compared with the control group, exenatide significantly reduced albuminuria after 24 weeks of intervention [149].

Chronic kidney disease mainly affects elderly populations worldwide, and it is a growing concern [150]. AWARD-7 trial investigated the glycemic control and kidney outcomes of dulaglutide in patients with T2DM and moderate-to-severe CKD. Dulaglutide was associated with less eGFR decline than insulin glargine at 52 weeks of follow-up [151]. The post-hoc analysis of SUSTAIN 1-7 trials showed that semaglutide decreases UACR [152]. Cardiovascular trails have shown that GLP-1 RAs can benefit CKD patients [153, 154]. A meta-analysis evaluated seven cardiovascular studies of GLP-1 RAs, stating that GLP-1 RAs treatment can reduce broad composite kidney outcomes, including the sustained decline in eGFR, emergence of new macroalbuminuria, progression to end-stage kidney disease (ESKD), or death from renal causes [154]. GLP-1 RAs reduce the risk of serious renal events compared with other hypoglycemic drugs, such as sulfonylureas and DPP-4 inhibitors [155, 156]. However, GLP-1 is less effective in preventing the progression of kidney diseases than SGLT-2 inhibitors in some cases [157]. The KDIGO (Kidney Disease: Improving Global Outcomes) 2020 clinical practice guidelines recommend that patient preferences, comorbidities, eGFR, and cost should be considered when approaches of choosing hypoglycemic drugs other than SGLT-2 inhibitors and metformin, if needed GLP-1 RAs are preferred, especially for the T2DM and CKD patients with an eGFR <30 ml/min per 1.73 m^2^ or those treated with dialysis [158]. The findings illustrate that GLP-1 RAs can improve DKD and CKD management.

In people over 60 years, the incidence of community-acquired AKI has increased by more than 3-fold [159]. Risk factors for AKI in this population include aging-related hemodynamic alterations, kidney aging, comorbidities and medications [140]. A few cases of GLP-1 RAs-induced AKI have been reported in middle-aged and elderly patients [160-162]. However, it has been reported that GLP-1 RAs are not correlated to the enhanced risk for AKI. It was found that the AKI rate was similar in liraglutide and placebo groups in LEADER trial [9]. Additionally, exenatide did not raise the incidence of acute renal failure (ARF) [163]. It has also been documented that SGLT2 inhibitors exert a lower risk for AKI than GLP-1 RAs [164]. However, the roles of GLP-1 RAs in AKI development among the elderly have not been fully established.

### 4. 5 The roles of GLP-1 RAs in musculoskeletal degenerative diseases

Musculoskeletal degenerative diseases, such as osteoporosis, osteoarthritis and sarcopenia are considerable health challenges among the elderly. The protective roles of GLP-1 RAs on osteoporosis have been elaborated in some detail.

GLP-1 receptor levels decreased significantly in rat models of knee osteoarthritis, while liraglutide inhibited the expression of inflammation-related proteins through the PKA/CREB signaling pathway [165]. Liraglutide attenuated cartilage degeneration in rat models [166]. At the cellular level, the GLP-1 receptor mediates the protective effects of liraglutide in chondrocytes, preventing endoplasmic reticulum stress and apoptosis [166]. In addition, liraglutide exerted beneficial effects on human chondrocytes by inhibiting oxidative stress, mitigating inflammatory responses and suppressing extracellular matrix degradation [167]. These results suggest the clinical potential of GLP-1 RAs in treating osteoarthritis.

Sarcopenia is associated with an aging-related progressive decline of muscle mass, quality, strength and function. In C2C12 myoblasts, liraglutide induced myogenesis through GLP-1 receptor and downstream cAMP-dependent pathways [168]. Additionally, the disrupted structure of myofibrillar was restored by liraglutide in some muscle atrophy models [168]. Analogously, exendin-4 and dulaglutide increases muscle mass and function through the suppression of myostatin and muscle atrophic factors and enhancement of myogenic factors [169]. Moreover, a clinical study showed liraglutide treatment was associated with improved skeletal muscle index (SMI) [71]. Nevertheless, current research on the relationship between GLP-1 RAs and sarcopenia are limited.

## 5. Prospective therapeutic applications

The elderly are susceptible to various diseases, while GLP-1 RAs have multiple pleiotropic effects. GLP-1 RAs have the potential to be used or can be used in the treatment of various aging-related diseases, including T2DM, overweight or obesity, hypertension, hyperlipidemia, atherosclerosis, vascular aging, kidney disease, AD, PD, osteoporosis, osteoarthritis and sarcopenia. In addition, hepatocyte senescence-associated hepatic fat accumulation and steatosis should be evaluated [170]. This is because GLP-1 RAs have shown various benefits in managing non-alcoholic fatty liver disease (NAFLD), alleviating hepatic inflammation, metabolic dysfunction, insulin resistance and lipotoxicity [171-173]. With the continued decline in physical functioning, loss of capacity and socioeconomic status, the elderly are more likely to experience isolation, loneliness, and consequent psychological problems. In this respect, GLP-1 RAs can improve cognitive functions in patients with mood disorders [174]. Therefore, GLP-1 RAs are potential adjunctive antidepressants. However, although GLP-1 RAs are safe and well-tolerated in most cases, given the higher rates of gastrointestinal adverse effects in GLP-1 RAs treated patients, their clinical administrations in elderly patients should be carefully and comprehensively considered. The therapeutic results, pharmacokinetic characteristics, administration dosage, frequency, and duration vary for different types of GLP-1 RAs. It is essential to determine the specific roles of each drug and their combined actions with other agents. Clinical trials should be performed to investigate the roles of GLP-1 RAs in elderly patients with multimorbidities, thus maximizing their efficacies while minimizing their side effects.

## 6. Perspectives and conclusions

GLP-1 RAs can safely and effectively lower blood sugar levels and reduce weight as a new hypoglycemic or weight loss drug. We have reviewed the diverse mechanisms underlying the protective function of GLP-1 RAs against aging-related diseases, especially in middle-aged and aged adults. Several hallmarks of aging have all been elucidated systematically and comprehensively. We strive to study the molecular, phenotypic, and functional hierarchical domains, establishing the interlaced time and hierarchical relationship of aging. Plenty of clinical evidence mainly focusing on middle-aged and aged adults support the established or potential benefits of GLP-1 RAs on a variety of common aging-related diseases, including metabolic diseases, neurodegenerative diseases, cardiovascular diseases, kidney diseases, and musculoskeletal degenerative diseases. Nevertheless, the complex mechanisms underlying the relationship between GLP-1 RAs and aging-related diseases have not yet been fully identified. Although GLP-1 RAs have great potential for a wide range of human diseases, there are many hurdles to bring them to the clinic. Large-scale studies are needed to verify the safety and efficacies of GLP-1 RAs in decreasing polypharmacy in elderly patients with multimorbidities.
